# Cell separation: Terminology and practical considerations

**DOI:** 10.1177/2041731412472690

**Published:** 2012-12-28

**Authors:** Matthew J Tomlinson, Sophie Tomlinson, Xuebin B Yang, Jennifer Kirkham

**Affiliations:** 1 Department of Oral Biology, Leeds Dental Institute, University of Leeds, Leeds, UK; 2 NIHR Leeds Musculoskeletal Biomedical Research Unit, Chapel Allerton Hospital, Leeds, UK

**Keywords:** cell separation, cell sorting, terminology, cell clusters, dead cells, tissue preparation

## Abstract

Cell separation is a powerful tool in biological research. Increasing usage, particularly within the tissue engineering and regenerative medicine communities, means that researchers from a diverse range of backgrounds are utilising cell separation technologies. This review aims to offer potential solutions to cell sorting problems and to clarify common ambiguities in terminology and experimental design. The frequently used cell separation terms of ‘purity’, ‘recovery’ and ‘viability’ are discussed, and attempts are made to reach a consensus view of their sometimes ambiguous meanings. The importance of appropriate experimental design is considered, with aspects such as marker expression, tissue isolation and original cell population analysis discussed. Finally, specific technical issues such as cell clustering, dead cell removal and non-specific antibody binding are considered and potential solutions offered. The solutions offered may provide a starting point to improve the quality of cell separations achieved by both the novice and experienced researcher alike.

## Introduction

Cell separation is a powerful tool, which is widely used in many strands of biological and biomedical research and in clinical therapy. For research, the ability to sort cells into distinct populations enables the study of individual cell types isolated from a heterogeneous starting population without (or with greatly reduced) contamination from other cell types. This technology underpins many discoveries in cell biology and is further enabling research in areas as diverse as regenerative medicine, cancer therapy and HIV pathogenesis.^1–3^

In terms of clinical usage, therapeutic cell separation allows for the introduction of enriched cell populations to a patient with a clinical need for those cells, for example, separation of leukocytes by aphaeresis or enrichment of haematopoietic stem cells by immunomagnetic separation.^4,5^ It also enables the enumeration of cells within an individual’s blood system and can aid repopulation of the immune system, for example, in multiple sclerosis patients who have undergone immunoablation treatment.^6^

Currently, most regenerative treatments based on cell separation are restricted to tissues such as blood and bone marrow.^5,7^ Recently, however, advances in stem cell therapy, tissue engineering and regenerative medicine are showing the potential for clinical cell-based therapies using cells derived from a variety of tissues, such as adipose and intestine.^8,9^ The use of highly selective cell separation procedures in clinical cell-based treatments has the potential to improve the quality of repair and the subsequent clinical outcome. Because of this potential, there is an increasing usage of these methodologies in the fields of tissue engineering and regenerative medicine, which has resulted in an increasing number of researchers using, or wanting to use, cell separation technologies. These researchers are drawn from a diverse range of backgrounds, not all of whom are necessarily based in biology. Indeed, the increasing demand for cell separation in multiple disciplinary research fields is not restricted to tissue engineering and regenerative medicine; cell sorting is also being used in many other areas such as biochemistry, electrical engineering, physics and materials science.^10–13^

A multitude of cell separation techniques currently available to researchers are based on three core themes: density, adherence and antibody binding, with many points of crossover between these different themes. New techniques incorporating microfluidics combined with a variety of cellular properties are also in development. Despite the differences between different cell separation techniques, they share common problems and pitfalls, which can at best hinder research progress and at worst give rise to erroneous data. Many of these technical problems and pitfalls are only applicable to certain techniques, whereas others are universal regardless of the method of separation. Other difficulties can arise in the experimental planning stage, where there can be a lack of understanding in identifying appropriate controls. Finally, there is a potential lack of clarity in the terminology used around cell separation methods, which can lead to confusion and a misunderstanding of the analytical measures required.

This review is written taking cognisance of the diversity of backgrounds and expertise of those researchers wishing to use cell sorting methods. The aim is not to produce a detailed step-by-step guide for each methodology but to offer potential solutions when common difficulties arise and provide clarity in areas of ambiguity related to experimental preparation and terminology.

## Cell separation techniques

A large variety of cell separation methods are currently commercially available, these are predominantly based on three methodologies: adherence, density and antibody binding. New techniques are being developed that utilise microfluidic technologies and take advantage of a variety of cellular properties such as elasticity in response to acoustic waves and membrane polarisation in a non-uniform electric field.^14,15^ However, these techniques are mostly still experimental and not yet available commercially for research. The choice of separation method depends upon a variety of factors, and each methodology has benefits and drawbacks that affect its applicability in a given situation. In this section, we will briefly outline the three overall methodologies with specific examples of each.

### Adherence

**Figure 1.**
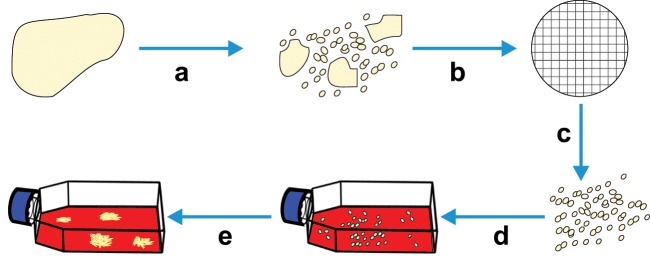
Diagram detailing cell separation by plastic adherence. (a) Whole tissue is disrupted into a cell suspension by enzymatic or mechanical means or a combination of both (separations of blood or bone marrow aspirate do not require this step). (b) Following disruption, the cells can be passed through a filter to remove cell clumps (c) giving a single-cell suspension, which will be added to (d) an adherent surface, and after a period of culture, (e) adherent cells can be observed.

Techniques that utilise cellular adherence are some of the most simple methods used for cell separation and are routinely used when isolating cells from digested or explanted primary tissues (Figure 1). An example of simple cell separation by adherence is the isolation of dental pulp stromal cells from whole digested dental pulp. In this technique, enzymatically digested dental pulp is filtered and plated directly onto tissue culture plastic, and following a period of culture, the adherent stromal cells are passaged.^16^ This technique benefits from being very simple and cheap, but it is not at all specific and relies on the cells of interest adhering and in some instances rapidly proliferating to outcompete other adherent cells in the suspension, such as neurons and monocytes. Adherence can also take time leading to some uncertainty as to the success of a separation. Recently, techniques based on cell adherence, such as differential binding of cells to polymer brushes of varying lengths, grafted to glass surfaces, have been developed and these are currently being refined.^17^ However, despite this progress, current uses of adherence sorting are mostly only applicable when cell purity is not of concern and isolation of various subpopulations is not required.

### Density

**Figure 2.**
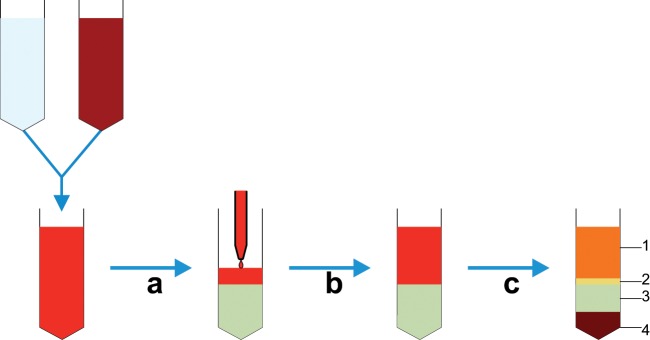
Diagram detailing whole blood cell separation by density gradient centrifugation. (a) Initially, whole blood is diluted with saline buffer, and (b) this is then carefully layered on top of the centrifugation medium contained in a conical tube avoiding any mixing of the two phases. (c) Following centrifugation, at the appropriate velocity without braking, distinct phases can be observed; 1 – plasma, 2 – interphase containing mononuclear cells, 3 – centrifugation medium and 4 – erythrocytes and granulocytes; cells can then be aspirated from the interphase.

**Figure 3.**
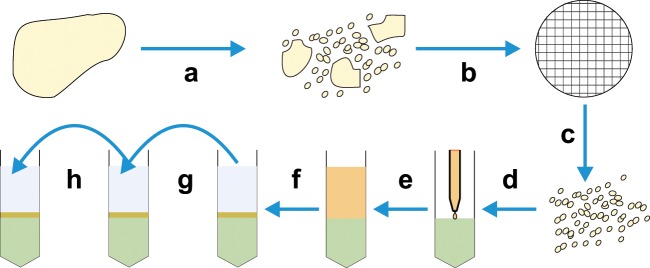
Diagram showing separation of solid tissue–derived cells by density gradient centrifugation. Tissues are (a) dissociated and (b) filtered to give (c) a single-cell suspension. (d) This suspension is carefully layered over a centrifugation medium avoiding mixing to give (e) two distinct phases, which can then be centrifuged to give (f) a cell-rich interphase between the centrifugation medium and the cell suspension buffer. (g and h) It is possible to isolate different cell fractions by removing cells from the supernatant or the interphase and then recentrifuging them at different concentrations of centrifugation medium and angular velocities until the desired fractions are obtained.

Density-based techniques are now mostly based on the use of centrifugation, although historically sedimentation-based methods have been employed.^18^ Techniques based on centrifugation are commonly used in many laboratories and are also routinely used clinically. The ability to sort large numbers of cells based on their density, relative to a graduated separation medium (usually sugar based), makes these techniques particularly applicable for separations involving the use of blood (Figure 2), which contains 4 × 10^9^ to 6.5 × 10^9^ cells/mL. Indeed, the most commonly used clinical cell separation method is aphaeresis of whole blood to isolate mononuclear cells for treatment of a variety of conditions, including leukaemia.^19^ However, despite the large-scale use of density-based methods, there are still problems with specificity as the differing densities of different cell populations are, in some instances, not large enough to be able to separate out individual cell types. These problems can be overcome by performing repeated centrifugations using differing concentrations of centrifugation medium and differing angular velocities. By using these techniques, it is possible to isolate different cell types from a complex mix, including disrupted solid tissues (Figure 3) such as mouse liver.^20^ However, although technically feasible, this is still challenging to perform with high specificity. As such, centrifugation methods are generally used if specificity is not absolutely necessary, as in aphaeresis, or as a pre-enrichment stage to remove cells like red blood cells and platelets.

Another density-based method used in laboratory separations is rosetting, which works as a combination between antibody binding and density methods. In this method, unwanted cells are labelled with antibodies that subsequently form complexes with erythrocytes, creating immunorosettes that are much denser than the mononuclear cells of interest. Following centrifugation, these rosettes, containing the labelled unwanted cells, pellet with erythrocytes leaving purified target cells in the mononuclear cell phase.^21^

Methods that sort cells by density are useful techniques to employ when working with tissues that contain a large number of unwanted cells, for example, blood, bone marrow and adipose tissue. This can be either for the isolation of a heterogeneous mix of cells, which can then be used experimentally, or as a pre-enrichment step prior to sorting by other methods.

### Antibody binding

**Figure 4.**
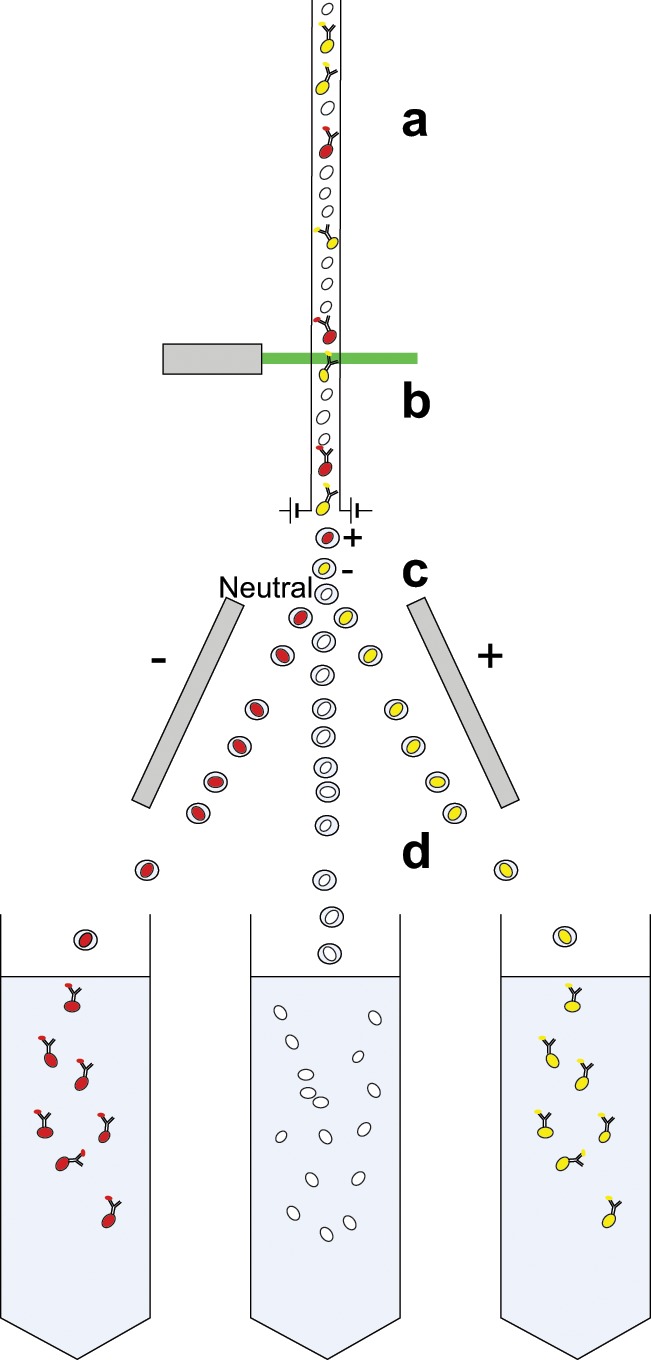
Diagram showing cell separation by FACS. Fluorescently labelled single cells from solid or fluid tissues, filtered to remove cell aggregates, are channelled to give a continuous stream of individual cells; (b) these cells then pass through a light source or laser, and the signature of each cell is detected. From this detection, the cells will be determined to be above or below a designated threshold value, and it is decided whether to collect or not collect each cell. (c) This is achieved by electrically charging the droplet each cell is contained within and (d) then by passing it through charged deflector plates that deflect the cells to the appropriate collection tubes. FACS: fluorescence-activated cell sorting.

**Figure 5.**
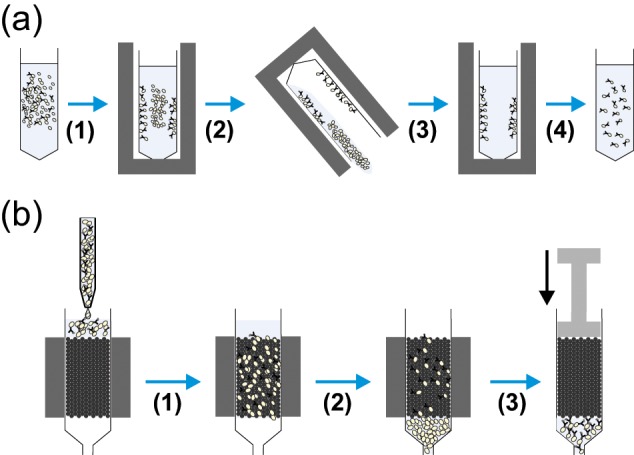
Diagrams showing the common methods used for magnetic cell separation. (a) Tube-based separation where a magnetically labelled cell suspension held in a conical tube is placed in a (1) magnet causing movement of labelled cells to the sides of the tube towards the magnet. This tube is then (2) inverted (or aspirated), allowing removal of the non-labelled cells before (3) resuspension of the labelled cells and removal from the magnet giving (4) a dispersed suspension of labelled target cells. (b) Column-based separation where a magnetically labelled cell suspension is injected into a column held within a magnet, (1) cells then flow through the column and (2) labelled cells are retained, whereas unlabelled cells are washed out. (3) Following the removal of unlabelled cells, the column is removed from the magnet, and suspension buffer is forced through the column by plunger giving labelled target cells in suspension.

Antibody-binding methods generally refer to the commonly used techniques of fluorescence-activated cell sorting (FACS) and magnetic-activated cell sorting (MACS).^22–24^ Both technologies utilise the same cellular properties for separation, namely, cell surface antigens against which antibodies are raised. FACS separation relies on the conjugation of fluorescent labels to these antibodies, whereas MACS uses conjugation to iron oxide containing microbeads. Following binding of conjugated antibodies, FACS and MACS proceed down different routes. FACS separation is achieved by laser excitation of the bound fluorophores, with excitation above a threshold level signalling the corresponding cell to be separated (Figure 4). MACS requires the cells to be placed in a magnetic field; unlabelled cells are eluted, and labelled cells are retained in the field until they are removed from the magnet, giving the separated populations (Figure 5). As such, a key difference between MACS and FACS is that MACS can be seen as a bulk method, there is no individual cell analysis, and magnetically tagged cells are retained and non-tagged cells are eluted. FACS, however, analyses each individual cell, which can be tagged with multiple antibodies, whereas MACS is restricted to individual markers (although some kits use enzymatic removal of the microbeads, allowing the cells to be relabelled with a subsequent antibody). This individual cell analysis means that while FACS can be more specific, it is significantly slower than MACS. Sorting that takes several hours by FACS can be achieved in less than 1 h by MACS.

There are other techniques, in addition to FACS and MACS, that utilise antibody binding to enable cell separation, an example of which is rosetting as previously mentioned. However, this is a relatively old technique, and there are many new technologies being developed, which use antibody or cell–ligand binding as the basis for separation. For example, antibodies, immobilised to polymer surfaces, have been used in a microfluidic system to capture circulating tumour cells from whole blood with subsequent release and enumeration.^25^ Columns have also been developed with antibody-immobilised surfaces to enrich osteoblastic cells based on CD34 binding.^26^ Polymer cryogels with large interconnected pores and surface-immobilised protein A ligands have been used to isolate antibody-labelled CD34^+^ umbilical cord blood cells in an affinity chromatography–based separation.^27^ Other methods in development include magnetophoresis,^28^ DNA aptamer binding^29^ and aqueous phase partitioning.^30^ However, despite the variety of antibody-based methods, for the purposes of this review, FACS and MACS will be focussed on due to the experimental nature of these newer techniques.

Antibody-based methods of separation are currently the gold standard for the selection of individual cell populations, and both FACS and MACS can be used to isolate cell populations to high purity. Despite this, there are still some problems with FACS and MACS such as the reliance on cell surface markers, which, for most researchers, limits separations to those markers for which antibodies are commercially available. It can also cause problems if the cell type of interest does not have unique markers, making the isolation of a homogeneous population difficult. For example, mesenchymal stem cells (MSCs) express markers associated with many other cell types such as CD90, which is also expressed by primitive haematopoietic stem cells.^31^ In addition, the isolation of a viable homogeneous population of cells that contain a unique intracellular marker can also be problematic, as the permeabilisation steps required to stain the marker can damage cell membranes leading to cell death.

### Lab-on-a-chip methods

In addition to the traditionally used methodologies for cell separation are several new lab-on-a-chip techniques that operate on a microfluidic scale and utilise a multitude of cellular characteristics to isolate different cell populations in a label-free manner. These techniques are mostly still in the experimental stage, but their development demonstrates the variety of possible ways to separate cells, and they are extensively reviewed by Gossett et al.^32^ Examples of label-free separation are the use of micro-scale filters or pillars that separate cells based upon size and membrane deformability, as larger cells are prevented from navigating through the filter leading to cell separation.^33^ Field flow fractionation (FFF) can be used to separate cells along the length of a microfluidic channel by a combination of the parabolic flow within the channel and an external field, such as an electric field or gravity. With FFF, particles that are more greatly affected by the external field are forced closer to the channel wall, which is moving more slowly than the centre of the channel and contains more weakly affected particles. Therefore, cell separation occurs because of the effect of the force on the cells and the speed of elution based on the cells’ location in the microfluidic channel.^34^ Acoustophoresis separates cells based on membrane deformation or elasticity and occurs when a high-pressure sound wave interacts with a cell. This interaction can cause membrane deformation to differing degrees based on the cell’s density and size and leads to the cells being positioned in different parts of the microfluidic channel and therefore able to be separated.^14^ Dielectrophoresis can lead to cell separation due to the differential polarisation of particles within a non-uniform electric field. This dipole effect depends on factors such as size and protein content and leads to the attraction or repulsion of the cell away from or towards an electrode. Due to differences in these factors between different cells, it is therefore possible to exert different effects on different cell types within the same field and allow for cell separation.^15^

Label-free lab-on-a-chip isolation methods have great potential to improve cell sorting methods both in a research environment and clinically. However, there are still potential problems associated with these techniques, many of which are general cell sorting problems, which can be applied to the commonly used techniques such as cell clusters, and others that are technique specific. One of the largest problems these techniques currently face is resolving the differences between cell types; for example, with dielectrophoresis, it can be difficult to discern the differences between target and non-target cells. However, perhaps the greatest challenge these techniques face is showing great enough efficacy while overcoming the challenges associated with currently used methods.

Overall, the choice of cell separation methodology is very much dependent upon the initial cell source, the characteristics of the desired cell type and its required purity. Adhesion-based techniques are useful if there is little requirement other than the isolation of adherent cells, and the cell of interest will, if necessary, outcompete other cell types. Centrifugation techniques are useful when dealing with samples with large cell numbers, such as blood, but where specificity is not essential, and are also useful as a pre-enrichment step prior to other separation methods. Antibody-mediated separation methods are the gold standard techniques currently available as they can be used to isolate specific cell populations. However, speed can be an issue, as can costs. Potentially, lab-on-a-chip methods will overcome some of the limitations in the currently used techniques, but, as yet, these are experimental and not accessible to the majority of the researchers performing cell sorting.

## Clinical cell therapy

The majority of separations currently performed for clinical cell therapy use cells isolated from tissues such as bone marrow and blood. These separations isolate the mononuclear cells, including the stem cell fraction, and can be used to recapitulate the haematopoietic system of a patient suffering from, for example, chronic myeloid leukaemia, following immune ablation therapy.^6^ These separations mostly utilise systems based on centrifugation, such as aphaeresis, as these technologies allow for the isolation of the large numbers of mononuclear cells needed for cell transplantation relatively quickly. MACS can also be used for cell therapy, and the clinically approved MACS-based systems use the same technology as research-grade magnetic sorting; however, these systems are closed and use reagents and fluidic tubing produced under good manufacturing practice (GMP) conditions.^35^ Use of MACS for clinical cell sorting allows for greater specificity than can be achieved by centrifugation; however, per patient, MACS is more expensive than aphaeresis, and so it is used in circumstances where specificity of the isolated cells is important.

Standard FACS-based systems are not in clinical use for cell therapy, although some flow cytometers can be used for clinical diagnostics.^36^ This is in part due to the difficulty in developing single-use sterile fluidics, the possibility of cross-contamination should multiuse fluidics be employed and problems with batch-to-batch consistency. There are currently methods utilising closed system optical separation in development, but these are not yet in widespread clinical usage.^37^

Clinical cell separation is an established field, but it has strict requirements, and there are challenges and difficulties to overcome. The major requirement is to ensure that a consistent, sterile cell population is isolated. Microbial contamination of cell separation products could lead to the infection of the recipient patient, who, in many instances, will be immunocompromised and unable to fight the infection. It is therefore imperative that clinical cell separation products are produced under strict GMP conditions with stringent batch testing. Consistency of the isolated cell population is also very important so as to ensure that the recipient receives the required cell transplant. In addition, rigorous tissue typing should be performed prior to transplantation to avoid human leukocyte antigen (HLA) mismatch and prevent problems such as graft-versus-host disease.

At this time, the major challenge for clinical cell separation is the robust isolation of rare cell populations with multiple surface markers from a large initial pool of cells. Currently, technologies based on centrifugation allow for the isolation of cells from a large initial cell number, and technologies based on MACS can isolate specific populations of cells; however, these technologies use single markers meaning that cells of interest with two or more markers cannot be specifically isolated. Development of high-speed optical cell sorters holds great promise, as these systems could have the speed of an MACS-based system, but with the specificity of an FACS system allowing for more than one parameter to be selected.

## Terminology and interpretation

When assessing the efficacy of any cell separation, there are three main considerations to take into account: purity, recovery and viability. Each of these apparently straightforward terms can be ambiguous when used in the context of cell separation and have been described so previously.^38^ This ambiguity can lead to confusion and difficulties in understanding the data following cell separation. An attempt to update, clarify and define each term is given below.

### Purity

Purity relates to the enrichment of cells of interest from a heterogeneous population based on known factors, such as cell surface phenotype, which are associated with those cells. From this separated fraction, the percentage of target cells compared to isolated non-target cells can be calculated. The word ‘associated’ is used in the definition above, as many such characteristics are related to more than one cell type. For example, the CD4 cell surface glycoprotein is strongly associated with T lymphocytes and is also expressed by regulatory T cells (Tregs) and some monocytes.^39,40^ Indeed, the CD4 T lymphocyte population itself is heterogeneous for several different T-cell subsets such as Th1 and Th2 T-helper cells, memory T cells, naive T cells and effector T cells.^41^ Therefore, unless the characteristic being selected for is specific to the cell type of interest, ‘purity’ can be an imprecise term when discussing enrichment. Using CD4 as an example, a separation could yield a 99% ‘pure’ population of CD4 positive cells, but within this population, there could be a heterogeneous mix of monocytes, Tregs and T lymphocyte subsets at different developmental stages. This difficulty also highlights the importance of a thorough understanding of the original cell population upon which to base the selection criteria. This will be discussed further in section ‘Considerations for experimental design’ below.

Given the difficulties in defining what ‘purity’ means in the context of a population of separated cells, it may be more appropriate to qualify purity as ‘target cell purity’ removing the reliance on the separation characteristic and indicating the need for awareness of the specific characteristics of the target cell. This, nonetheless, maintains the use of the word ‘purity’, which remains contentious as it implies homogeneity, a term that is difficult to apply to any population of cells due to cell-specific differences in, for example, cell cycle and differentiation. It might therefore be more helpful to use a term relating to the enrichment or depletion of the target cells when defining the outcome of a cell separation.

### Recovery

‘Recovery’ is usually used to describe the percentage of cells that are obtained post sorting compared to the number of total cells or target cells in the original suspension. As such, the recovery, in conjunction with the purity, gives information on the efficiency of the cell separation. There are two potential measurements that can be made relating to recovery: separated cells versus total cell count and separated cells versus target cells in the original cell suspension, which is generally more informative. The former measurement will yield information on the percentage of cells, which have been isolated from the total number of cells, and will provide guidance on the separation efficiency when working with a cell suspension with a well-defined composition. However, the value of this measurement is limited, especially for original cell suspensions with a variable content due to, for example, a disease state. To determine the true separation efficiency, the number of recovered cells must be compared to the number of target cells in the original suspension. It is therefore important to perform analysis on both the cells obtained following separation and the original cell suspension.

‘Recovery’ in the context of cell separation is therefore ambiguous. Both values described above provide information on recovery, but one details the *overall* recovery, and the other details the recovery of the cells of interest. It might therefore be more appropriate to distinguish the two terms as ‘total recovery’ and ‘target recovery’. Total recovery is useful when a quick figure is needed for a well-defined population such as peripheral blood mononuclear cells from healthy donors, but when there is uncertainty over the constituents of a population, such as in a disease state where population percentages change such as sepsis, target recovery is a much more informative term.^42^

### Viability

The final descriptor of cell sorting efficacy, ‘viability’, again requires clarification. At its most basic level, viability can be taken to mean ‘cells that are not dead’. This descriptor is clearly important as a separation process that does not yield live cells is of little value when the downstream application is a live cell assay or cells for clinical applications. However, the fact that cells are alive does not of itself necessarily meet with the requirements for these applications, for example, senescent cells are also live but do not possess the capability to proliferate or differentiate.^43^ Such changes or transformations would impair the cell’s ability to function in a representative or in vivo manner in in vitro studies and need to be avoided. Therefore, viability in its strictest definition is only an indicator of cell quality after separation; live cell assays will not provide information about cells that are pre-apoptotic, senescent or incapable of differentiation. To measure these parameters, other methodologies must be used such as resazurin for metabolic activity, bromodeoxyuridine (BrdU) for proliferation and in the case of stem cells, colony forming capability.^44–46^ These assays give a more relevant assessment of the ability of the cells to be used in downstream applications, taking into consideration additional factors other than whether the cells are merely alive. It is therefore important that, in addition to assessing viability, the user determines whether the isolated cells are fit for the purpose by performing additional assays.

Taken together, ‘purity’, ‘recovery’ and ‘viability’ appear at face value to be a straightforward set of measurements to perform on separated cells, but there is greater depth to these parameters than is first apparent. If detailed analyses of the purity, recovery and viability are performed, then a picture of the quality of a cell separation can be built up. This not only can provide information on an individual separation but can also be used to validate a separation technique or validate the applicability of a cell source to an individual technique.

## Considerations for experimental design

Initial planning and design is key for any experimental strategy, including cell separation, where many factors must first be considered. These factors impact different stages of the separation procedure, but all share a basic set of preliminary requirements. These are the need for a detailed understanding of the cell and tissue types of interest, knowledge of the potential techniques available and the ability to select the correct methodology to yield the desired cell population.

The reason for this required level of understanding is that one cell separation method may be more suitable than another for achieving a given outcome, and different cells react differently to the same conditions. Current methods for cell separation generally offer a balance between purity and recovery. It is therefore important that the separation protocol is designed with this in mind and tailored to suit the desired outcome. For example, if a large number of cells are required, then percentage enrichment may need to be sacrificed; alternatively, for a highly enriched population, the trade-off may be low numbers recovered. Factors to be considered when designing a cell separation strategy are discussed below.

### Cost

Cost is a design constraint that is relevant to most separation experiments. Cell separation can be a potentially expensive technology depending on the strategy selected. It may therefore be important to devise a strategy that is not prohibitively expensive by employing cost-saving measures. For example, FACS is a very accurate technique, but it can be slow when sorting rare cells from whole blood, and this consequently increases the running time on the instrument and thus the expense. A way of reducing this time would be to perform an initial erythrocyte lysis step or density gradient centrifugation to remove the erythrocytes, leaving only the mononuclear cells to sort.^47^ Pretreatment of a sample can thus reduce overall cost and should be considered where cost is an issue.

### Receptor stimulation

One of the most important prerequisites for successful experimental design is a detailed knowledge of the cell type of interest. This is illustrated by antibody-mediated cell separation, where knowledge of cell surface receptors is very important. The reason for this is that some cell surface receptors, such as CD3, are stimulated upon antibody binding leading to their internalisation with resulting changes in the cell phenotype.^48^ If the aim of a study was to investigate these changes, it would clearly be desirable to avoid initiating them during the separation stage itself. In this case, it would be advisable to use a strategy whereby all other cell types are depleted leaving the enriched cell type of interest untouched. This separation strategy would then yield enriched cells that have had minimal antibody stimulation and which could then be studied further, the trade-off being a reduction in recovery of the desired cells.

### Receptor cleavage

Knowledge of the specific characteristics of the cell of interest is also important to ensure that appropriate pre-sorting sample preparation procedures are employed to maximise recovery of that cell type. For example, when dissociating cells from solid tissue, the choice of enzyme is key because certain cell surface glycoproteins such as CD4 and CD133 can be cleaved by (or are predicted to be cleaved by) trypsin.^49,50^ A detailed knowledge of the biology of the cell and its receptors, therefore, reduces the potential for such marker cleavage and, therefore, the potential for false negatives.

### Receptor expression

Another potential confounding issue with selection based upon surface markers is their possible weak expression and downregulation, again leading to false negatives. Examples of these include CD19 and CD271.^51,52^ CD19 is a marker used for the isolation of B cells, but it is not strongly expressed compared to other common lymphocyte markers, such as CD4 and CD8, which have antibody-binding capacities of over four and six times greater than CD19, respectively.^51,53,54^ Any cell sorting technique that utilises receptor binding, such as immunomagnetic separation, may therefore quickly lead to receptor saturation. This makes downstream analysis difficult as the epitope is no longer available to be bound by fluorochrome-conjugated antibodies, resulting in false negatives by flow cytometry. This issue can be overcome by utilising CD20 as an alternative marker for mature B cells and leukaemic B cells, and this demonstrates the requirement of a detailed knowledge of B-cell surface molecules.^55–57^ Another consequence of low-level receptor expression is subsequent low antigen binding capacities, meaning applications such as immunomagnetic separation may not be as efficient, leading to the requirement to enrich target cells by depleting non-target cells.^58^

An example of a surface marker that is down regulated is CD271 (also known as low-affinity nerve growth factor receptor), a positive marker for MSCs isolated from several human tissue sources, including bone marrow.^59^ CD271 is expressed on the cell surface of bone marrow MSCs immediately following isolation and can be used to select for an MSC population. One of the most common methods of MSC isolation is plastic adherence, where the MSC population is included in the cells that adhere to the culture plate plastic. However, this leads to CD271 being downregulated, and as such, CD271 can no longer be used to identify the MSC subset.^52^ Alternative markers such as CD105 and CD73 must therefore be used, again highlighting the need for detailed knowledge of the target cell’s biology.^60^

### Original fraction aliquot

The examples above show the importance of a thorough knowledge of cell surface markers and, indeed, of the cell population in general when designing a cell sorting experimental strategy. A thorough working knowledge of the original cell population is also key to understanding and interpreting data around recovery and enrichment and hence the overall efficiency of a cell separation. It is therefore important to always remove an aliquot of the original cell sample immediately prior to separation as this gives a snapshot of the cells before sorting commenced. This is also important when working with cells from patients with diseases affecting cellular composition, such as sepsis, where expected percentages of cellular subsets can be dramatically different from the accepted norm.^42^ Having information on the original fraction allows for an accurate assessment of the separation efficiency at its culmination by flow cytometric comparison using both the positive and negative fractions.

### Tissue preparation

The final and perhaps most important consideration for experimental design prior to cell sorting is the time taken for the sample to be prepared following excision and the storage conditions of that sample. Speed of process from tissue to sorted cells is a very important factor to consider when designing an experiment because of cell death over time. Cell viability will begin to fall soon after isolation due to a variety of factors such as enzymatic action and nutrient limitations if the sample is left at ambient temperature.^61^ Many previous studies such as those by Caviedes-Bucheli et al.^62^ on dental pulp and D’Armini et al.^63^ on lung show that if tissue remains in this state for an extended period of time (greater than 24 h), then significant cell death will occur. Previous work has also indicated that for tissue to be considered viable, it must have less than 50% dead cells, which has implications for downstream studies.^64^ This cell death may affect the quality of the subsequent separation due to dead cell–mediated cell–cell binding and non-specific antibody binding and will necessitate dead cell removal.^65^ Therefore, it is preferable to work with tissue that has been excised immediately prior to beginning any cell separation.

Effective methods are available to reduce necrosis in excised tissue for short periods where necessary. These are mainly based on temperature, although use of appropriate transport media and oxygenation of the sample can also improve tissue quality prior to dissociation. Tissue maintenance at 4°C is an effective short-term measure to reduce necrosis, although this only delays the degradation and is ineffective over periods greater than 24 h.^66^ One alternative that is becoming increasingly effective is freezing whole tissue for separation at a later date. This requires effective cryopreservation to reduce cell shearing due to ice crystal growth, and it can still result in a high degree of cell death. Techniques to reduce this are improving steadily, but it is still only recommended when tissue processing within 24 h is not possible.^67,68^

Choice of an appropriate transport media can significantly improve viability in isolated tissue and cells, with certain media such as Euro-Collins solution and L15M15 medium improving cell viability compared to phosphate-buffered saline.^69,70^ Increasing numbers of tissue storage solutions, such as those that mimic interstitial fluid, are becoming available.^71^ These solutions are designed to improve whole organ transplantation, but as they improve cell viability, they will also benefit cell separation applications.^72^ The choice of media is a tissue-specific consideration with different tissues and cells requiring different media, which is beyond the scope of this review. Oxygenation of media is another means of improving tissue viability in some tissues, for example, human pancreas in cold storage.^73^

It is worth mentioning that the points above are generic and not tissue specific; each tissue must be considered according to its individual merits and requirements. For example, whole blood may require the use of an anticoagulant, such as heparin, whereas bone marrow does not.^74^ It is for the user to determine the most appropriate preparatory steps prior to separation. What is common to all tissue types, however, is the fact that even the very best and most efficient cell sorting technique available will always be limited by the quality of the starting sample.

## Resolution of methodological difficulties

**Figure 6.**
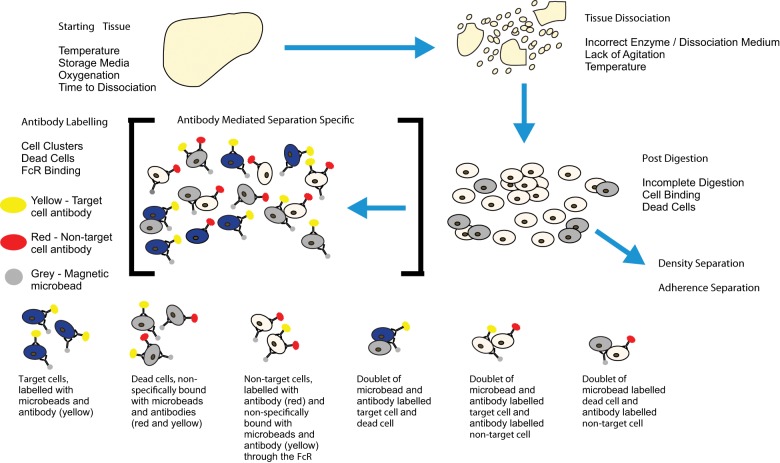
Diagram showing an overview of the potential problems at each stage of cell separation: starting tissue, tissue dissociation, post-digestion and antibody labelling. Blue cells are target cells, beige cells are non-target cells and grey cells are dead. Highlighted are some of the potential problems caused by non-specific antibody binding and dead cells.

There are several key technical considerations that must be taken into account before performing a successful cell separation, some of which are universally applicable, while others are more specific to immunomagnetic and immunofluorescent cell separation. Figure 6 gives an overview of potential technical problems at each stage during the separation process.

The more universal considerations relate to the quality of the cells, which are being separated, and specifically to the cell isolation process. Antibody-mediated separations also have considerations relating to antibody binding. There can also be specific idiosyncratic problems associated with different commercially available cell separation products such as incubation temperature and supernatant removal, but it is not within the remit of this review to discuss these. Any specific technical issues is best dealt with by the company responsible for the product.

Cell isolation and preparation are essential prerequisites when sorting cells but can be the cause of many technical difficulties when resulting suspensions contain clusters of cells and/or a high proportion of dead cells. For the purposes of this review, we are defining a cell cluster as an association of two or more cells. Cell clusters can arise when working with both solid tissue- and blood-derived cells due to incomplete dissociation or post-dissociative association/aggregation. The presence of cell clusters can result in reduction in isolated cell purity due to co-isolation of non-target cells that are conjoined with the cells of interest or loss of target cells due to their binding with cells that are removed from the suspension as part of the separation process. On a practical level, and especially in the case of flow sorting, some immunomagnetic separations and microfluidic-based techniques, such as dielectrophoresis and FFF, clusters of cells can also block fluid flow and prevent the subsequent separation of remaining cells by preventing them from entering the system.

### Tissue dissociation and cell clusters

The causes of cell clusters are often incomplete dissociation (mainly via enzymatic digestion) of the tissue or re-establishment of cell–cell contacts between the dissociated cells. In the case of blood, coagulation can cause clustering. Clusters can also be related to dead cells due to release of DNA.^75^ Apoptotic and pre-apoptotic cells release DNA due to chromosomal fragmentation and necrotic cells by macrophage-mediated action.^76^ Extracellular DNA has been shown to induce cell aggregation, and it is thought that this is due to DNA-binding domains on the cell surface.^77,78^ Fortunately, methods for circumventing these pitfalls are relatively simple and easy to implement. Initial tissue disruption by mechanical methods, in general, can improve enzyme digestion due to the increased surface area of disrupted tissue, therefore reducing both the time required for dissociation and potential enzyme-induced cell death.^79,80^ Ensuring an efficient enzymatic digestion is often dependent upon the choice of enzyme used. The large number of enzymes available for tissue dissociation is beyond the scope of this review; however, most enzymatic digestions can be enhanced by utilising rotation, providing continuous agitation of the suspension.^81^ This movement can improve both the speed and quality of the digestion reducing the incidence of cell clustering and cell death in a given cell suspension. Other simple methods to ensure achievement of a single-cell suspension include passing the cells through a filter with a pore size of between 30 and 70 µm, thus removing the vast majority of cell clusters, and suspending cells at densities below 1 × 10^6^ cells/mL to reduce the potential for aggregation. However, if the initial digestion is inefficient, this will lead to a potential loss of target cells.

In order to prevent dissociated cells reattaching and forming clusters, it can be beneficial to resuspend cells in buffer without magnesium and calcium ions and containing a chelator, such as ethylenediaminetetraacetic acid (EDTA) to sequester calcium ions that are used in cell–cell contacts.^82^ This inhibits cells binding and helps maintain a single-cell suspension. In addition to a chelator, the addition of DNase I can reduce clusters by digesting DNA released by dead cells, which can also lead to cell binding.^75^

### Dead cells

Dead cells can be a significant problem with cell separation, and as a general rule, if there are more than 10% dead cells in a given suspension, then steps must be taken to remove them. Below this level, their effect on subsequent separation is, while not ideal, usually acceptable. The reason for the need to remove dead cells from suspension is twofold: (a) dead cells release DNA that aggregates cells and (b) dead cells can bind non-specifically to antibodies leading to false positives.^83^ The first problem can be resolved by the addition of DNase I as suggested previously, reducing the potential for aggregation. In the second case, while non-specific antibody binding is not restricted to dead cells, it is nevertheless a problem that is strongly associated with them. The problem of dead cells is exacerbated in the case of antibody-mediated cell separation because non-target dead cells will be labelled along with the target population leading to a reduction in the purity of the isolated cells. Binding can be reduced by the addition of a blocking reagent to occupy potential non-specific binding sites, but a more effective method is to remove the dead or dying cells completely with a pre-separation step. There are many different means of identifying dead cells. The more common techniques include labelling with DNA intercalating dyes such as TO-PRO-3, propidium iodide, trypan blue, ethidium homodimer and 7-AAD, and binding of annexin V to phosphatidylserine and labelling with amine reactive dyes such as ViViD.^84–90^ Flow sorting and immunomagnetic separation are the most common methods to then remove these labelled dead and dying cells, but other techniques are being developed utilising microfluidics and dielectrophoresis to achieve the same depletion.^91,92^

### FcR binding

Non-specific antibody binding is not only associated with dead cells, but other factors, such as antibody binding to the fragment, crystallisable region-receptor (FcR), excess of antibody and use of indirectly conjugated antibodies and immunomagnetic particles, can also result in non-specific labelling of cells.

Antibody binding to the FcR is a common cause of non-specific binding, generating false positives in antibody-mediated cell separation in consequence. The FcR is a cell surface receptor that recognises the Fc component of antibodies and is part of the immunoglobulin superfamily found on cells of the immune system.^93^ The resulting recognition event results in formation of an antibody–receptor complex that stimulates an immune response.^94^ The FcR is different to the other immune system receptors (i.e. T-cell and B-cell receptors) in several ways because it is located on several different haematopoietic cell types such as monocytes and macrophages.^95^ The other major difference from other receptors is that the FcR recognises antibody, not antigen, recognising all classes of antibody.^93,96^ FcR–antibody binding leads to the problem of non-specific binding due to FcRs on non-target haematopoietic-derived cells binding free antibody.

The problem of FcR-derived non-specific antibody labelling can cause significant issues with false positives but is relatively straightforward to overcome. FcR blocker is commercially available for use when labelling cells. This blocking reagent is species-specific and binds to the FcR, preventing antibody binding. Blocking reagents can be synthetic or natural; synthetic reagents consist of peptides directed against the FcR, and natural reagents are IgG molecules or antibodies such as anti-CD16 or anti-CD32, which bind to and block the FcR preventing non-specific binding.

## Summary and discussion

Currently available strategies for cell separation can yield highly enriched cell suspensions. However, there are potential problems that can impair the overall quality of the separation, and these need to be recognised by the increasingly interdisciplinary user base and addressed where they arise. In addition, experimental planning and terminology need to be carefully considered.

In the initial experimental design and planning stages, it is important to understand what outcomes are going to be assessed, that is, how are purity, recovery and viability being measured? It is important to identify the characteristic which purity is being measured against, which population the recovered cells are being compared to and which measure of viability is being assessed. If these terminological ambiguities are defined prior to beginning the experimental regimen, it can make identifying technical problems easier.

A thorough knowledge of the cell suspension and the cell type to be isolated can vastly improve the quality of a separation. This is particularly important for cell separation based on antibody binding. It is also important to collect samples at key points during the separation process so that the efficiency of the separation can be assessed. This point is related to the terminological considerations, as these data are required to determine the purity, recovery and viability. Perhaps the most important preparatory step prior to separation is the storage of the starting tissue. The key aspect with this is speed of tissue processing, with dissociation and cell separation immediately following tissue excision being greatly preferred. If this is not possible, then various means can be employed to reduce tissue necrosis, the most important of which is temperature.

Technically, there are several factors that can compromise the quality of a cell separation and subsequently the overall data acquired. These factors can be distilled down to two main problems: clusters of cells and false-positive cell sorting. Both of these problems have multiple causes, some of which overlap. The general problems are incomplete tissue digestion, re-establishment of cell–cell contacts, release of DNA by dying cells, non-specific antibody labelling of dead cells and non-specific antibody binding to the FcR. None of these technical problems are insurmountable, but they can lead to significant problems without knowledge and awareness of the issues together with appropriate measures taken to address them.

Overall, it is hoped that this review clarifies terminology, provides guidance to experimental set-up and gives reasons for and solutions to potential problems that can arise during the process of cell separation. We hope that we have achieved our aim of providing the user with an understanding of why certain terminology is used and what it means, why certain aspects of planning and set-up are key to successful separations and what the main technical difficulties that can arise during the process are and how they can be resolved.

## References

[1] Guo KT Schafer R Paul A A new technique for the isolation and surface immobilization of mesenchymal stem cells from whole bone marrow using high-specific DNA aptamers. Stem Cells 2006; 24: 2220–2231 16794266 10.1634/stemcells.2006-0015

[2] Takaishi S Okumura T Tu SP Identification of gastric cancer stem cells using the cell surface marker CD44. Stem Cells 2009; 27: 1006–1020 19415765 10.1002/stem.30 PMC2746367

[3] Terry VH Johnston ICD Spina CA CD44 microbeads accelerate HIV-1 infection in T cells. Virology 2009; 388: 294–304 19394995 10.1016/j.virol.2009.03.022 PMC2699902

[4] Handgretinger R Lang P Schumm M Isolation and transplantation of autologous peripheral CD34(+) progenitor cells highly purified by magnetic-activated cell sorting. Bone Marrow Transplant 1998; 21: 987–993 9632271 10.1038/sj.bmt.1701228

[5] To LB Haylock D Simmons PJ The biology and clinical uses of blood stem cells. Blood 1997; 89: 2233–2258 9116266

[6] Mancardi G Saccardi R Autologous haematopoietic stem-cell transplantation in multiple sclerosis. Lancet Neurol 2008; 7: 626–636 18565456 10.1016/S1474-4422(08)70138-8

[7] Stamm C Westphal B Kleine H-D Autologous bone-marrow stem-cell transplantation for myocardial regeneration. Lancet 2003; 361: 45–46 12517467 10.1016/S0140-6736(03)12110-1

[8] Zuk PA Zhu M Ashjian P Human adipose tissue is a source of multipotent stem cells. Mol Biol Cell 2002; 13: 4279–4295 12475952 10.1091/mbc.E02-02-0105 PMC138633

[9] Lanzoni G Alviano F Marchionni C Isolation of stem cell populations with trophic and immunoregulatory functions from human intestinal tissues: potential for cell therapy in inflammatory bowel disease. Cytotherapy 2009; 11: 1020–1031 19929466 10.3109/14653240903253840

[10] Ackerman SJ Liu L Kwatia MA Charcot-Leyden crystal protein (galectin-10) is not a dual function galectin with lysophospholipase activity but binds a lysophospholipase inhibitor in a novel structural fashion. J Biol Chem 2002; 277: 14859–14868 11834744 10.1074/jbc.M200221200

[11] Howard D Partridge K Yang X Immunoselection and adenoviral genetic modulation of human osteoprogenitors: in vivo bone formation on PLA scaffold. Biochem Biophys Res Commun 2002; 299: 208–215 12437971 10.1016/s0006-291x(02)02561-5

[12] Yang J Huang Y Wang XJ Dielectric properties of human leukocyte subpopulations determined by electrorotation as a cell separation criterion. Biophys J 1999; 76: 3307–3314 10354456 10.1016/S0006-3495(99)77483-7 PMC1300300

[13] Chan JW Taylor DS Lane SM Nondestructive identification of individual leukemia cells by laser trapping Raman spectroscopy. Anal Chem 2008; 80: 2180–2187 18260656 10.1021/ac7022348

[14] Petersson F Åberg L Swärd-Nilsson A-M Free flow acoustophoresis: microfluidic-based mode of particle and cell separation. Anal Chem 2007; 79: 5117–5123 17569501 10.1021/ac070444e

[15] Hu X Bessette PH Qian J Marker-specific sorting of rare cells using dielectrophoresis. Proc Natl Acad Sci U S A 2005; 102: 15757–15761 16236724 10.1073/pnas.0507719102 PMC1276091

[16] Gronthos S Mankani M Brahim J Postnatal human dental pulp stem cells (DPSCs) in vitro and in vivo. Proc Natl Acad Sci U S A 2000; 97: 13625–13630 11087820 10.1073/pnas.240309797 PMC17626

[17] Nagase K Kimura A Shimizu T Dynamically cell separating thermo-functional biointerfaces with densely packed polymer brushes. J Mater Chem 2012; 22: 19514–19522

[18] Miller RG Phillips RA Separation of cells by velocity sedimentation. J Cell Physiol 1969; 73: 191–201 5783244 10.1002/jcp.1040730305

[19] Buckner D Graw RG Eisel RJ Leukapheresis* by continuous flow centrifugation (CFC) in patients with chronic myelocytic leukemia (CML). Blood 1969; 33: 353–369 5250470

[20] Liu W Hou Y Chen H Sample preparation method for isolation of single-cell types from mouse liver for proteomic studies. Proteomics 2011; 11: 3556–3564 21751380 10.1002/pmic.201100157

[21] Strelkauskas AJ Teodorescu M Dray S Enumeration and isolation of human T and B lymphocytes by rosette formation with antibody-coated erythrocytes. Clin Exp Immunol 1975; 22: 62–71 813929 PMC1538335

[22] Bonner WA Sweet RG Hulett HR Fluorescence activated cell sorting. Rev Sci Instrum 1972; 43: 404–409 5013444 10.1063/1.1685647

[23] Miltenyi S Müller W Weichel W High gradient magnetic cell separation with MACS. Cytometry 1990; 11: 231–238 1690625 10.1002/cyto.990110203

[24] Rembaum A Yen RCK Kempner DH Cell labeling and magnetic separation by means of immunoreagents based on polyacrolein microspheres. J Immunol Methods 1982; 52: 341–351 7130709 10.1016/0022-1759(82)90006-0

[25] Adams AA Okagbare PI Feng J Highly efficient circulating tumor cell isolation from whole blood and label-free enumeration using polymer-based microfluidics with an integrated conductivity sensor. J Am Chem Soc 2008; 130: 8633–8641 18557614 10.1021/ja8015022 PMC2526315

[26] Mahara A Yamaoka T Continuous separation of cells of high osteoblastic differentiation potential from mesenchymal stem cells on an antibody-immobilized column. Biomaterials 2010; 31: 4231–4237 20185169 10.1016/j.biomaterials.2010.01.126

[27] Kumar A Srivastava A Cell separation using cryogel-based affinity chromatography. Nat Protoc 2010; 5: 1737–1747 21030950 10.1038/nprot.2010.135

[28] Schneider T Karl S Moore LR Sequential CD34 cell fractionation by magnetophoresis in a magnetic dipole flow sorter. Analyst 2010; 135: 62–70 20024182 10.1039/b908210g PMC3509203

[29] Xu Y Phillips JA Yan JL Aptamer-based microfluidic device for enrichment, sorting, and detection of multiple cancer cells. Anal Chem 2009; 81: 7436–7442 19715365 10.1021/ac9012072 PMC3164879

[30] Sousa AF Andrade PZ Pirzgalska RM A novel method for human hematopoietic stem/progenitor cell isolation from umbilical cord blood based on immunoaffinity aqueous two-phase partitioning. Biotechnol Lett 2011; 33: 2373–2377 21858668 10.1007/s10529-011-0727-0

[31] Baum CM Weissman IL Tsukamoto AS Isolation of a candidate human hematopoietic stem-cell population. Proc Natl Acad Sci U S A 1992; 89: 2804–2808 1372992 10.1073/pnas.89.7.2804 PMC48751

[32] Gossett DR Weaver WM Mach AJ Label-free cell separation and sorting in microfluidic systems. Anal Bioanal Chem 2010; 397: 3249–3267 20419490 10.1007/s00216-010-3721-9 PMC2911537

[33] Ji HM Samper V Chen Y Silicon-based microfilters for whole blood cell separation. Biomed Microdevices 2008; 10: 251–257 17914675 10.1007/s10544-007-9131-x

[34] Vykoukal J Vykoukal DM Freyberg S Enrichment of putative stem cells from adipose tissue using dielectrophoretic field-flow fractionation. Lab Chip 2008; 8: 1386–1393 18651083 10.1039/b717043b PMC2726253

[35] Lang P Schumm M Taylor G Clinical scale isolation of highly purified peripheral CD34+ progenitors for autologous and allogeneic transplantation in children. Bone Marrow Transplant 1999; 24: 583–589 10490722 10.1038/sj.bmt.1701961

[36] Brown M Wittwer C Flow cytometry: principles and clinical applications in hematology. Clin Chem 2000; 46: 1221–1229 10926916

[37] Digiusto DL Cooper LJN Preparing clinical grade Ag-specific T cells for adoptive immunotherapy trials. Cytotherapy 2007; 9: 613–629 17943498 10.1080/14653240701650320 PMC2238678

[38] Sharpe PT Methods of cell separation. Amsterdam: Elsevier Science, 1988, p. 272

[39] Lucey DR Dorsky DI Nicholsonweller A Human eosinophils express CD4 protein and bind human immunodeficiency virus-1 gp120. J Exp Med 1989; 169: 327–332 2783333 10.1084/jem.169.1.327 PMC2189179

[40] Groux H Ogarra A Bigler M A CD4(+) T-cell subset inhibits antigen-specific T-cell responses and prevents colitis. Nature 1997; 389: 737–742 9338786 10.1038/39614

[41] Mosmann TR Sad S The expanding universe of T-cell subsets: Th1, Th2 and more. Immunol Today 1996; 17: 138–146 8820272 10.1016/0167-5699(96)80606-2

[42] Williams RC Koster FT Kilpatrick KA Alterations in lymphocyte cell-surface markers during various human infections. Am J Med 1983; 75: 807–816 6605684 10.1016/0002-9343(83)90412-6

[43] Hayflick L The limited in vitro lifetime of human diploid cell strains. Exp Cell Res 1965; 37: 614–636 14315085 10.1016/0014-4827(65)90211-9

[44] Castro-Malaspina H Gay R Resnick G Characterization of human bone marrow fibroblast colony-forming cells (CFU-F) and their progeny. Blood 1980; 56: 289–301 6994839

[45] Seifalian AM Salacinski HJ Punshon G A new technique for measuring the cell growth and metabolism of endothelial cells seeded on vascular prostheses. J Biomed Mater Res 2001; 55: 637–644 11288093 10.1002/1097-4636(20010615)55:4<637::aid-jbm1058>3.0.co;2-z

[46] Crissman HA Steinkamp JA A new method for rapid and sensitive detection of bromodeoxyuridine in DNA-replicating cells. Exp Cell Res 1987; 173: 256–261 2960553 10.1016/0014-4827(87)90350-8

[47] Bøyum A Isolation of lymphocytes, granulocytes and macrophages. Scand J Immunol 1976; 5: (Suppl. 5): 9–15 1052391

[48] Chatenoud L Bluestone JA CD3-specific antibodies: a portal to the treatment of autoimmunity. Nat Rev Immunol 2007; 7: 622–632 17641665 10.1038/nri2134

[49] Rao PE Talle MA Kung PC Five epitopes of a differentiation antigen on human inducer T cells distinguished by monoclonal antibodies. Cell Immunol 1983; 80: 310–319 6192938 10.1016/0008-8749(83)90119-3

[50] Sakariassen PO Immervoll H Chekenya M Cancer stem cells as mediators of treatment resistance in brain tumors: status and controversies. Neoplasia 2007; 9: 882–892 18030356 10.1593/neo.07658 PMC2077879

[51] Ginaldi L De Martinis M Matutes E Levels of expression of CD19 and CD20 in chronic B cell leukaemias. J Clin Pathol 1998; 51: 364–369 9708202 10.1136/jcp.51.5.364 PMC500695

[52] Battula VL Treml S Bareiss PM Isolation of functionally distinct mesenchymal stem cell subsets using antibodies against CD56, CD271, and mesenchymal stem cell antigen-1. Haematologica 2009; 94: 173–184 19066333 10.3324/haematol.13740 PMC2635396

[53] Nadler LM Anderson KC Marti G B4, a human lymphocyte-B-associated antigen expressed on normal, mitogen-activated, and malignant lymphocytes-B. J Immunol 1983; 131: 244–250 6408173

[54] Lenkei R Andersson B Determination of the antibody binding capacity of lymphocyte membrane antigens by flow cytometry in 58 blood donors. J Immunol Methods 1995; 183: 267–277 7602149 10.1016/0022-1759(95)00064-h

[55] Loken MR Shah VO Dattilio KL Flow cytometric analysis of human-bone marrow 2. Normal lymphocyte-B development. Blood 1987; 70: 1316–1324 3117132

[56] Stashenko P Nadler LM Hardy R Characterization of a human lymphocyte-B-specific antigen. J Immunol 1980; 125: 1678–1685 6157744

[57] Greaves MF Hariri G Newman RA Selective expression of the common acute lymphoblastic-leukemia (gp100) antigen on immature lymphoid-cells and their malignant counterparts. Blood 1983; 61: 628–639 6338969

[58] McCloskey KE Moore LR Hoyos M Magnetophoretic cell sorting is a function of antibody binding capacity. Biotechnol Prog 2003; 19: 899–907 12790655 10.1021/bp020285e

[59] Jones EA Kinsey SE English A Isolation and characterization of bone marrow multipotential mesenchymal progenitor cells. Arthritis Rheum 2002; 46: 3349–3360 12483742 10.1002/art.10696

[60] Dominici M Le Blanc K Mueller I Minimal criteria for defining multipotent mesenchymal stromal cells. The International Society for Cellular Therapy position statement. Cytotherapy 2006; 8: 315–317 16923606 10.1080/14653240600855905

[61] Laiho K Penttilä A Autolytic changes in blood cells and other tissue cells of human cadavers. I. Viability and ion studies. Forensic Sci Int 1981; 17: 109–120 7239364 10.1016/0379-0738(81)90003-7

[62] Caviedes-Bucheli J Avendaño N Gutierrez R Quantification of lactate-dehydrogenase and cell viability in postmortem human dental pulp. J Endod 2006; 32: 183–185 16500222 10.1016/j.joen.2005.10.040

[63] D’Armini AM Tom EJ Roberts CS When does the lung die? Time course of high energy phosphate depletion and relationship to lung viability after ‘Death’. J Surg Res 1995; 59: 468–474 7564319 10.1006/jsre.1995.1193

[64] O’Driscoll SW Meisami B Miura Y Viability of periosteal tissue obtained postmortem. Cell Transplant 1999; 8: 611–616 10701490 10.1177/096368979900800607

[65] Clarke C Davies S Immunomagnetic cell separation. In: Brooks SA Schumacher U (eds) Metastasis research protocols. New York: Humana Press, 2001, pp. 17–23

[66] McNally RT Brockbank KGM Issues surrounding the preservation of viable allograft heart valves. J Med Eng Technol 1992; 16: 34–38 1640446 10.3109/03091909209021955

[67] Reuther T Kochel M Mueller-Richter U Cryopreservation of autologous bone grafts: an experimental study on a sheep animal model. Cells Tissues Organs 2010; 191: 394–400 20051679 10.1159/000273267

[68] Chan SCW Lam SKL Leung VYL Minimizing cryopreservation-induced loss of disc cell activity for storage of whole intervertebral discs. Eur Cell Mater 2010; 19: 273–283 20533193 10.22203/ecm.v019a26

[69] Dreikorn K Horsch R Rohl L 48- to 96-hour preservation of canine kidneys by initial perfusion and hypothermic storage using the Euro-Collins solution. Eur Urol 1980; 6: 221–224 6993210 10.1159/000473336

[70] Kischer CW Leibovitz A Pindur J The use of a transport medium L14M15 for bulk tissue storage and retention of viability. Cytotechnology 1989; 2: 181–186 22358732 10.1007/BF00133243

[71] Ozeki T Kwon MH Gu JY Heart preservation using continuous ex vivo perfusion improves viability and functional recovery. Circ J 2007; 71: 153–159 17186994 10.1253/circj.71.153

[72] Kibondo A Talbot D Cullis-Hill D Perfusates: their properties and usage for the maintenance and storage of organs for transplantation. Curr Anaesth Crit Care 2010; 21: 216–219

[73] Ricordi C Fraker C Szust J Improved human islet isolation outcome from marginal donors following addition of oxygenated perfluorocarbon to the cold-storage solution. Transplantation 2003; 75: 1524–1527 12792508 10.1097/01.TP.0000058813.95063.7A

[74] Geissmann F Jung S Littman DR Blood monocytes consist of two principal subsets with distinct migratory properties. Immunity 2003; 19: 71–82 12871640 10.1016/s1074-7613(03)00174-2

[75] Renner WA Jordan M Eppenberger HM Cell-cell adhesion and aggregation: influence on the growth behavior of CHO cells. Biotechnol Bioeng 1993; 41: 188–193 18609537 10.1002/bit.260410204

[76] Pisetsky DS Fairhurst A-M The origin of extracellular DNA during the clearance of dead and dying cells – review. Autoimmunity 2007; 40: 281–284 17516210 10.1080/08916930701358826

[77] Steinberg MS ‘ECM’: its nature, origin and function in cell aggregation. Exp Cell Res 1963; 30: 257–279 13983727 10.1016/0014-4827(63)90299-4

[78] Hefeneider SH McCoy SL Morton JI DNA binding to mouse cells is mediated by cell-surface molecules: the role of these DNA-binding molecules as target antigens in murine lupus. Lupus 1992; 1: 167–173 1301977 10.1177/096120339200100308

[79] Klein AB Witonsky SG Ahmed SA Impact of different cell isolation techniques on lymphocyte viability and function. J Immunoassay Immunochem 2006; 27: 61–76 16450869 10.1080/15321810500403755

[80] Marthinuss J Andrade-Gordon P Seiberg M A secreted serine protease can induce apoptosis in Pam212 keratinocytes. Cell Growth Differ 1995; 6: 807–816 7547502

[81] Noel JS Zucker RM Wu NC The dissociation of transplantable tumors. J Histochem Cytochem 1977; 25: 544–553 197162 10.1177/25.7.197162

[82] Booth C O’Shea JA Potten CS Maintenance of functional stem cells in isolated and cultured adult intestinal epithelium. Exp Cell Res 1999; 249: 359–366 10366435 10.1006/excr.1999.4483

[83] Stewart CC Stewart SJ Multiparameter analysis of leukocytes by flow cytometry. Methods Cell Biol 1994; 41: 61–79 7861980 10.1016/s0091-679x(08)61709-4

[84] Tennant JR Evaluation of the trypan blue technique for determination of cell viability. Transplantation 1964; 2: 685–694 14224649 10.1097/00007890-196411000-00001

[85] Van Hooijdonk CAEM Glade CP Van Erp PEJ TO-PRO-3 iodide: a novel HeNe laser-excitable DNA stain as an alternative for propidium iodide in multiparameter flow cytometry. Cytometry 1994; 17: 185–189 7530620 10.1002/cyto.990170212

[86] Nicoletti I Migliorati G Pagliacci MC A rapid and simple method for measuring thymocyte apoptosis by propidium iodide staining and flow cytometry. J Immunol Methods 1991; 139: 271–279 1710634 10.1016/0022-1759(91)90198-o

[87] Papadopoulos NG Dedoussis GVZ Spanakos G An improved fluorescence assay for the determination of lymphocyte-mediated cytotoxicity using flow-cytometry. J Immunol Methods 1994; 177: 101–111 7822816 10.1016/0022-1759(94)90147-3

[88] Schmid I Krall WJ Uittenbogaart CH Dead cell discrimination with 7-amino-actinomycin-D in combination with dual color immunofluorescence in single laser flow-cytometry. Cytometry 1992; 13: 204–208 1547670 10.1002/cyto.990130216

[89] Perfetto SP Chattopadhyay PK Lamoreaux L Amine reactive dyes: an effective tool to discriminate live and dead cells in polychromatic flow cytometry. J Immunol Methods 2006; 313: 199–208 16756987 10.1016/j.jim.2006.04.007

[90] Koopman G Reutelingsperger CPM Kuijten GAM Annexin-V for flow cytometric detection of phosphatidylserine expression on B-cells undergoing apoptosis. Blood 1994; 84: 1415–1420 8068938

[91] Shafiee H Sano MB Henslee EA Selective isolation of live/dead cells using contactless dielectrophoresis (cDEP). Lab Chip 2010; 10: 438–445 20126683 10.1039/b920590j

[92] Kose AR Fischer B Mao L Label-free cellular manipulation and sorting via biocompatible ferrofluids. Proc Natl Acad Sci U S A 2009; 106: 21478–21483 19995975 10.1073/pnas.0912138106 PMC2799875

[93] Fridman W Fc receptors and immunoglobulin binding factors. FASEB J 1991; 5: 2684–2690 1916092 10.1096/fasebj.5.12.1916092

[94] Daëron M Fc receptor biology. Annu Rev Immunol 1997; 15: 203–234 9143687 10.1146/annurev.immunol.15.1.203

[95] Ravetch JV Kinet JP Fc-receptors. Annu Rev Immunol 1991; 9: 457–492 1910686 10.1146/annurev.iy.09.040191.002325

[96] Heyman B Complement and Fc-receptors in regulation of the antibody response. Immunol Lett 1996; 54: 195–199 9052877 10.1016/s0165-2478(96)02672-7

